# Supplementary material for: Genome-wide identification and characterization of m6A regulatory genes in Soybean: Insights into evolution, miRNA interactions, and stress responses
Source: PLoS One. 2025 Jul 24;20(7):e0328773. doi: 10.1371/journal.pone.0328773 (PMC12289078; doi:10.1371/journal.pone.0328773)
Supplement: S1 Fig — All m6A regulatory genes are found to be located on different chromosomes of soybeans. The relative size of the corresponding chromosomes and the position of the respective genes could be estimated by using the scale provided left side of the figure. The chromosome numbers are provided in the middle of each bar. The straight lines connect the duplicated gene pairs. (PDF) [file pone.0328773.s001.pdf]

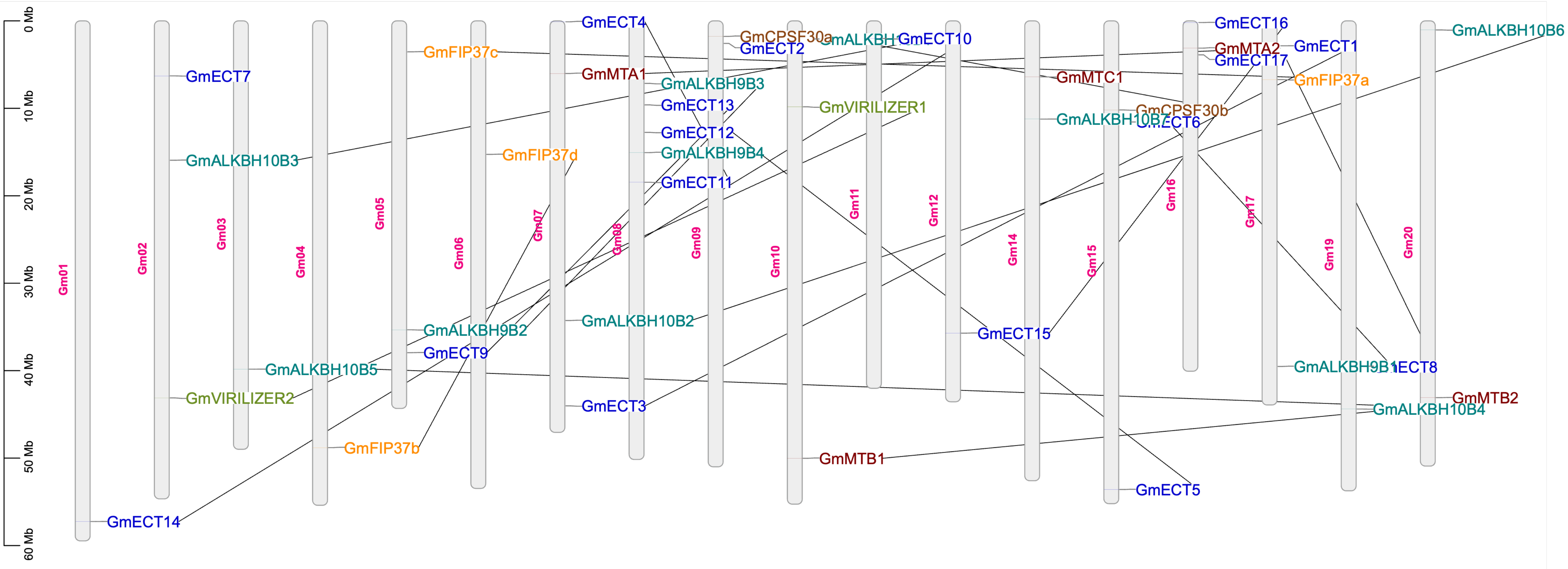

S1 Fig. Chromosomal distribution of m6A regulatory genes. All m6A regulatory genes are found to be located on different chromosomes of soybeans. The relative size of the corresponding chromosomes and the position of the respective genes could be estimated by using the scale provided left side of the figure. The chromosome numbers are provided in the middle of each bar. The straight lines connect the duplicated gene pairs.
