# Supplementary material for: Genome-wide identification and characterization of m6A regulatory genes in Soybean: Insights into evolution, miRNA interactions, and stress responses
Source: PLoS One. 2025 Jul 24;20(7):e0328773. doi: 10.1371/journal.pone.0328773 (PMC12289078; doi:10.1371/journal.pone.0328773)
Supplement: S2 Fig — The organization and distribution of the conserved motifs in the m6A writer (A), eraser (B), and reader (C) genes. The squares in the motif represent the positions of conserved domains. (PDF) [file pone.0328773.s002.pdf]

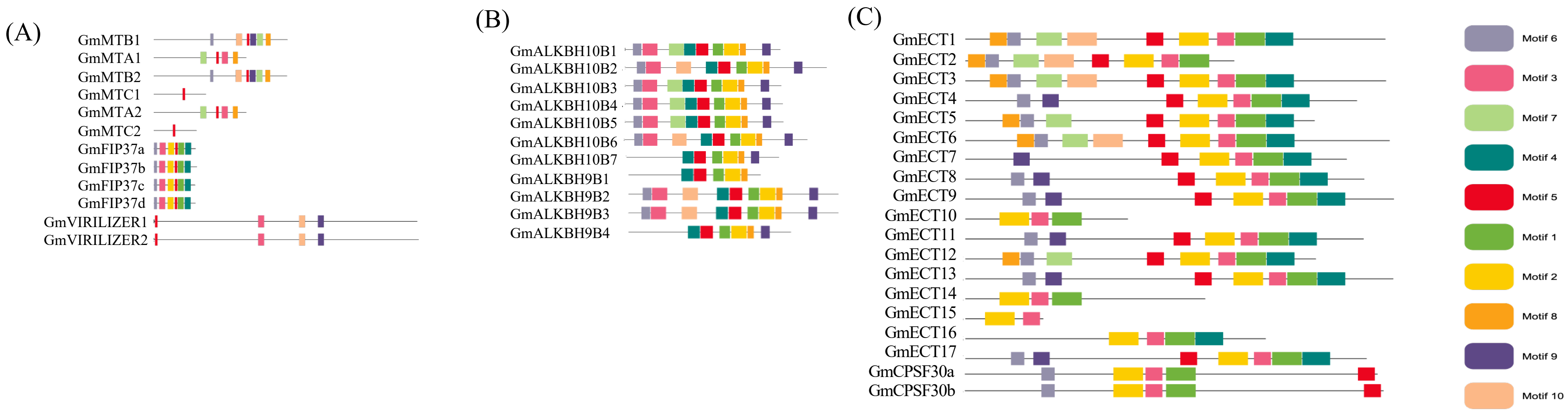

S2 Fig. Organization and distribution of the conserved motifs. The organization and distribution of the conserved motifs in the m6A writer **(A)**, eraser **(B),** and reader **(C)** genes. The squares in the motif represent the positions of conserved domains.
