# Supplementary material for: Genome-wide identification and characterization of m6A regulatory genes in Soybean: Insights into evolution, miRNA interactions, and stress responses
Source: PLoS One. 2025 Jul 24;20(7):e0328773. doi: 10.1371/journal.pone.0328773 (PMC12289078; doi:10.1371/journal.pone.0328773)
Supplement: S3 Fig — Two clusters (A and B) were identified using MCODE. C. The top 10 hub genes were extracted by the Density of Maximum Neighborhood Component method in the cytohubba plugin in Cytoscape. (PDF) [file pone.0328773.s003.pdf]

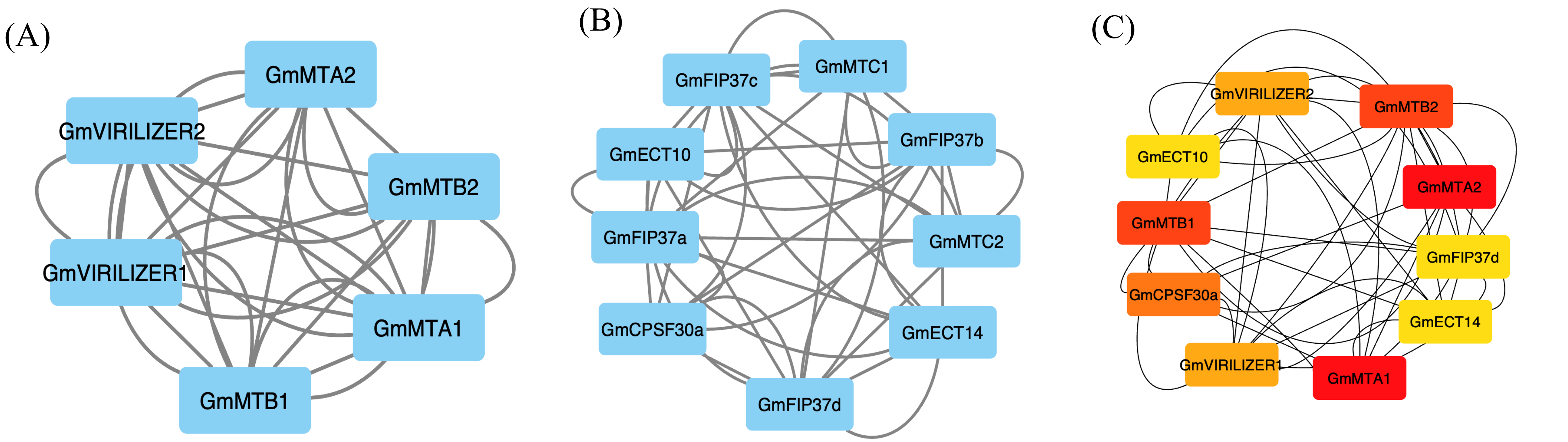

S3 Fig. Cluster and hub genes identification in soybeans. Two clusters **(A and B)** were identified using MCODE. **C.** The top 10 hub genes were extracted by the Density of Maximum Neighborhood Component method in the cytohubba plugin in Cytoscape.
