# Supplementary material for: Genome-wide identification and characterization of m6A regulatory genes in Soybean: Insights into evolution, miRNA interactions, and stress responses
Source: PLoS One. 2025 Jul 24;20(7):e0328773. doi: 10.1371/journal.pone.0328773 (PMC12289078; doi:10.1371/journal.pone.0328773)
Supplement: S4 Fig — Genome-wide miRNA-regulated networks of the writer (A), eraser (B), and reader (C). Green nodes: miRNAs, Pink nodes: genes that may be miRNA targets, and Black edges: correlations. (PDF) [file pone.0328773.s004.pdf]

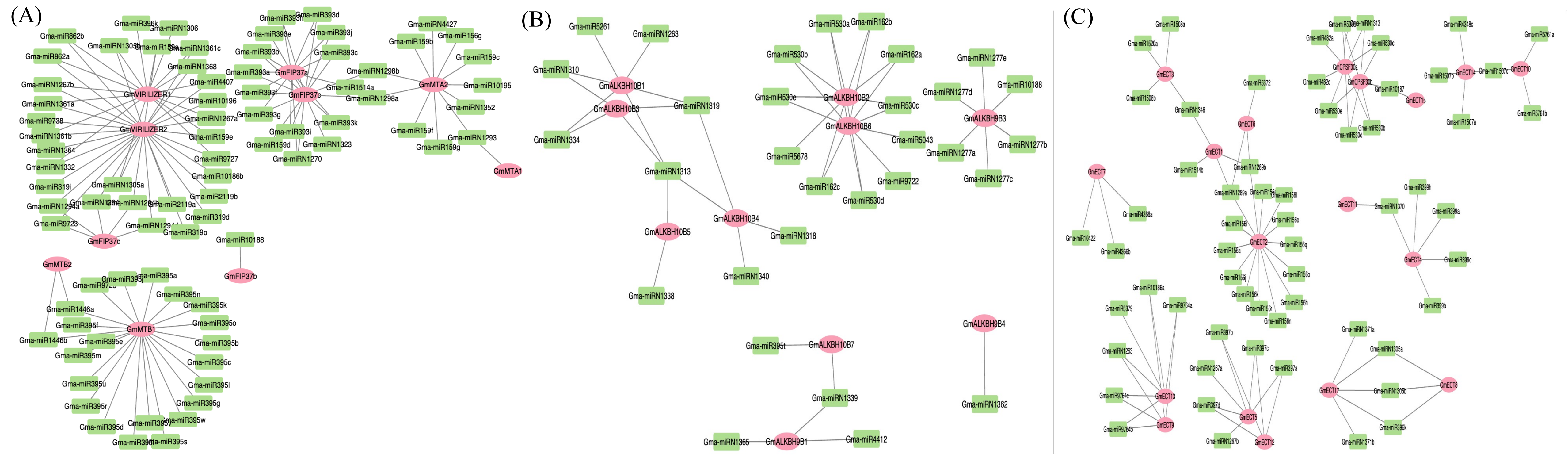

S4 Fig. Genome-wide network of soybean miRNA. Genome-wide miRNA-regulated networks of the writer **(A),** eraser **(B),** and reader **(C)**. Green nodes: miRNAs, Pink nodes: genes that may be miRNA targets, and Black edges: correlations.
